# Estimating the Standing Long Jump Length from Smartphone Inertial Sensors through Machine Learning Algorithms

**DOI:** 10.3390/bioengineering10050546

**Published:** 2023-04-29

**Authors:** Beatrice De Lazzari, Guido Mascia, Giuseppe Vannozzi, Valentina Camomilla

**Affiliations:** 1 Department of Movement, Human and Health Sciences, University of Rome “Foro Italico”, Piazza Lauro de Bosis 6, Lazio, 00135 Roma, Italy; b.delazzari@studenti.uniroma4.it (B.D.L.); g.mascia@studenti.uniroma4.it (G.M.); valentina.camomilla@uniroma4.it (V.C.); 2 Interuniversity Centre of Bioengineering of the Human Neuromusculoskeletal System, University of Rome “Foro Italico”, Piazza Lauro de Bosis 6, Lazio, 00135 Roma, Italy; 3 GoSport s.r.l., Via Basento, Lazio, 00198 Roma, Italy

**Keywords:** SLJ, IMU, accelerometer, prediction, in-field test

## Abstract

The length of the standing long jump (SLJ) is widely recognized as an indicator of developmental motor competence or sports conditional performance. This work aims at defining a methodology to allow athletes/coaches to easily measure it using the inertial measurement units embedded on a smartphone. A sample group of 114 trained young participants was recruited and asked to perform the instrumented SLJ task. A set of features was identified based on biomechanical knowledge, then Lasso regression allowed the identification of a subset of predictors of the SLJ length that was used as input of different optimized machine learning architectures. Results obtained from the use of the proposed configuration allow an estimate of the SLJ length with a Gaussian Process Regression model with a RMSE of 0.122 m in the test phase, Kendall’s τ < 0.1. The proposed models give homoscedastic results, meaning that the error of the models does not depend on the estimated quantity. This study proved the feasibility of using low-cost smartphone sensors to provide an automatic and objective estimate of SLJ performance in ecological settings.

## 1. Introduction

The standing long jump (SLJ) is a sports-related movement that requires complex motor coordination of both upper and lower body segments. It is a task used to evaluate both children’s motor competence [1], upper and lower body muscular fitness [2,3], and lower limbs muscular strength in sports-related field. In this last domain, the SLJ is often part of athletic training since it represents an explosive type of motor task. As a recognized functional test, it allows the analysis of the coordinated development of lower-body forces in the horizontal direction as a proxy for sprint performance in runners [4,5], which is also crucial in team sports such as football [6] or rugby [7,8,9]. The SLJ test is also used for several other aims: talent identification [7], prediction of player performance at different player positions [6], assessment of the efficacy of a training intervention [8,9], and anaerobic power prediction [10,11]. Its role as screening tool for athletes with increased injury risk was also investigated [12] together with its use to determine muscle imbalance [13]. Despite the wide potential for SLJ to evaluate performance, all these studies were limited to the meter-based assessment of the jumped distance as main parameter due to its simple and ecological evaluation. Only a few studies characterized the power expressed during the jump through direct measures with force platforms, having the aim of estimating anaerobic performance [10,11] or as biomechanical analyses to enhance the SLJ performance [14,15,16,17,18].

Several instrumented biomechanical analyses of the SLJ were performed to gain insight into motor behavior and coordination of both lower and upper body parts using force plates [14,15,16], optoelectronic motion capture [17,18], or inertial measurement units (IMUs) [1,19]. Of relevance is the analysis of the contribution of the arms to the motor behavior of the whole body, which determines an improvement in the jumped SLJ distance with respect to jumping without moving the arms [20,21]. Models developed in 2D and 3D suggest modeling the motion as planar when the interest is related only to lower body movement, while a 3D model can be useful when upper body motion is included [18].

While force plates and optoelectronic motion capture are expensive and constrained to the laboratory environment, IMUs are less expensive and can be used in an open field, eventually supervised by a tester. However, their cost still does not allow a large-scale democratic application of on-the-field instrumented tests [22,23]. A low-cost alternative seems to be the use of a smartphone (SP), which natively embodies IMUs. If hand-held, an SP could allow a fast biomechanical analysis of the lower limb movement independent of the presence of external testers. Although such embedded sensors were not developed specifically for biomechanical analysis, and therefore do not necessarily satisfy some required specifications such as high sampling frequency or appropriate full-scale range, the broad use of SP devices constitutes a beneficial alternative and could allow an evaluation in both laboratory and open-field environments from a biomechanical point of view [23].

Distance estimation based on magneto-inertial measurement units (MIMUs) can certainly rely on laws of motion and biomechanical models, as completed for height of countermovement jumps [24,25], but the poor quality of the available signals [23] has led to exploiting biomechanical features obtained from MIMUs as input to ad-hoc machine learning (ML) models in several sport applications [26,27,28]. Few ML approaches used SP data to assess jump-related variables only for the countermovement jump (CMJ): jump height [29], jump power [30,31], and fatigue [32]. To the best of our knowledge, only two studies contributed to developing ML approaches for SLJ: (i) the work of [16], which identified through Lasso regularization biomechanical variables measured with force plates as predictors for SLJ length; and (ii) the work of [2], which estimated the SLJ length using categorical and not categorical variables linked mainly to the anthropometric characteristics of the jumpers as input of generalized regression neural networks. The former model can have a limited diffusion due to the use of force platforms, while the latter, using only the jumper anthropometric characteristics, cannot explain the biomechanical variability of the jumper when performing more than one SLJ. To the best of our knowledge, no attempts have been performed exploiting either IMUs or SP-IMUs which could enable an ecological collection of informative biomechanical quantities both in laboratory and on-field.

The aim of this work is to use IMUs embedded in smartphones to estimate the SLJ length starting from non-categorical biomechanical features related to the jump technique and from the intrinsic anthropometric characteristics of the user. Biomechanical variables were selected based on two assumptions: (i) in the preparation phase, the SLJ is similar to the CMJ, presenting an eccentric and a concentric phase, thus sharing a similar behavior along the vertical acceleration; (ii) in the flight phase, the origin of the sensor coordinate system follows a parabolic trajectory. Six ML models dedicated to regression analysis were trained, optimized, and tested to this aim.

## 2. Materials and Methods

### 2.1. Experimental Setup

One hundred fourteen healthy sports science students were recruited as participants (79M, 35F; mean ± SD: age = 21.4 ± 5.1 years; stature = 1.8 ± 0.1 m; mass = 70.9 ± 10.3 kg). Only physically active healthy young sport science students were included, while individuals who underwent either lower limb surgery or injury in the six months prior to the experimental session were excluded from the study. All participants signed an informed consent prior to the experimental session. The study was approved by the local Internal Review Board.

**Figure 1:**
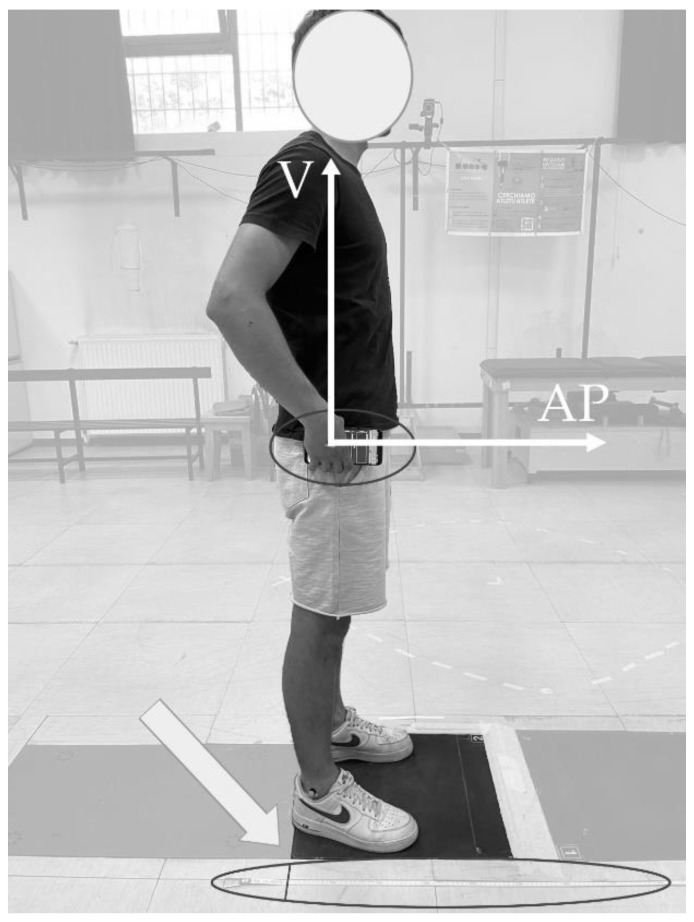
Experimental setup. The participant is in the static phase before jumping, following akimbo style, and holding the SP in the right hand and kept fixed with the hip (small black ellipse). The tape meter (big black ellipse) is located with the zero (highlighted with a black sign and a white arrow) near to the right heel, corresponding to the initial position.

Participants were equipped with an SP held in their right hand, as in Figure 1 (Samsung Galaxy S9+, Samsung Group, Seoul, South Korea; sampling frequency = 500 samples/s; full scale range: accelerometer = ±8 g; gyroscope = ±500 deg/s). All SP-IMU data were collected using the app Phyphox [33], which was remotely controlled through the laboratory PC. Sensor calibration tests were performed on the SP-IMU before each experimental session, as detailed in the “Data Processing” section. Afterwards, each participant was instructed on how to properly perform a SLJ and then performed 3 trials following the instructions of the operator. Jumps were executed with the left hand on the hip and the right one near to the hip while holding the SP horizontally (Figure 1). Holding the arms still permits the keeping of the SP in a stable position near to the hip, which is crucial to segment the jump in the three listed phases: (i) a static phase of a few seconds with the participant being with hands on the hips, feet in parallel stance position, and heels positioned at the zero of a meter tape; (ii) the jumping trial triggered by a vocal command; (iii) an after-landing second static phase. The jump was considered correct if the participant succeeded in maintaining the equilibrium after landing without realizing an additional step, keeping the feet in the parallel stance position and the arms still. The heel-to-heel distance, measured using the meter tape, was considered as the reference jump length to be estimated.

### 2.2. Data Processing

First, the SP-IMUs were calibrated before each experimental session for computing and eventually correcting their offset and cross-axis sensitivity according to [34]. Namely, the gyroscope static bias was obtained from a 60 s static trial with the SP still on a flat surface, subsequently removed from each successive jump measure. Concerning the accelerometer, three ad hoc 60 s static acquisitions were performed; each consisted in aligning one of the three accelerometer axes with the gravity vector direction [34]. To allow for a consistent gravity removal, acceleration measures were expressed into the global coordinate system under the hypothesis that the smartphone was kept parallel to the plane of movement and therefore not requiring an accurate estimate of its yaw [24]. The vertical (a_V_) and anteroposterior (a_AP_) components were then considered for further computations.

**Figure 2:**
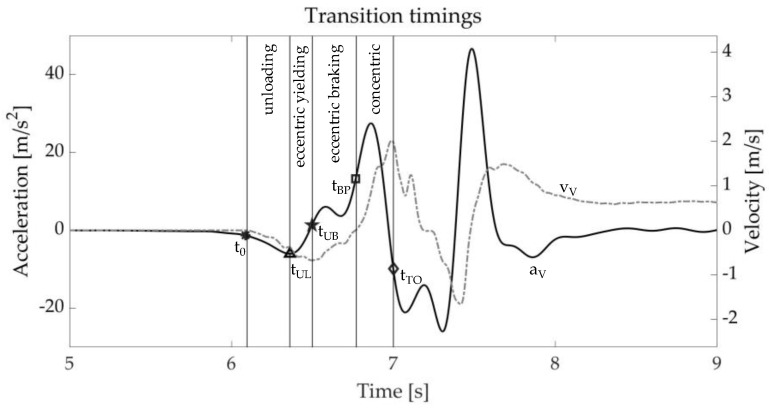
Vertical acceleration and velocity with highlighted transition timings and phases: unloading (between t_0_ and t_UL_), eccentric yielding (between t_UL_ and t_UB_), eccentric braking (between t_UL_ and t_BP_), and concentric propulsive phase (between t_BP_ and t_TO_). Legend for time instants: t_0_ = jump onset; t_a_min_ = minimum acceleration; t_a_max_ = maximum acceleration; t_v_min_ = minimum velocity; t_P_min_ = minimum power; t_P_max_ = maximum.

The preparation phase of the SLJ, similarly to the CMJ [35], can be subdivided in two phases: the eccentric and the concentric one. Further subphases were also considered in accordance with [16] (Figure 2): the unloading phase starts from the jump onset (t_0_) and arrives to the minimum of the vertical acceleration (t_UL_); the eccentric yielding phase is the time between the local minimum of the vertical acceleration and the minimum vertical velocity (t_UB_); the eccentric braking phase is defined as the time between the minimum vertical velocity and when that velocity crosses 0 (t_BP_); and concentric propulsive phase starts when the vertical velocity crosses 0 until take-off (t_TO_).

The vertical velocity, v_V_, was computed through numerical integration of the corresponding acceleration from the SLJ onset (t_0_) to take-off (t_TO_). The integration interval was kept to a minimum to limit the noise contribution due to integration drift. The delimiting time frames were computed as follows: the onset, t_0_, as the time sample occurring 30 ms prior the first one deviating by 8 times the standard deviation of the static phase, similarly to [36]; the take-off, t_TO_, as the first frame such that a_V_ ≤ −g. All data processing was performed using MATLAB R2022a (The MathWorks Inc., Natick, MA, USA).

### 2.3. Feature Selection

A total of F = 61 features, defined in Table 1, were extracted. Three were related to the anthropometric characteristics of the subject ((·)_anthro_ subscript): stature, body mass, and age. Four features were calculated from the acquired acceleration signals under the assumption that the trajectory of the origin of the sensor coordinate system during a SLJ can be approximated to a ballistic motion. Namely, the raw estimate of SLJ length (b_jump_), SLJ height (h_jump_), time of flight (t_flight_), and velocity angle at the take-off (α) were included. These values can be obtained from the vertical and antero-posterior velocities at take-off. Computing these velocity values requires only the identification of onset and take-off instants and the computation of the velocity time history in this time interval. The remaining 54 features were computed from either a_V,_ a_AP_, or both, as they similarly contribute to the SLJ distance estimate. Namely, 42 jump-related variables (features from A to R and ν, calculated twice where needed because they were evaluated for both V and AP components—(·)_V_ and (·)_AP_, respectively) were inspired by [37]; 6 (u, W, and z) enriched the biomechanical description of power-related variables as presented in [29]. The last six were time-frequency features obtained by processing a_V_ and a_AP_ via variational mode decomposition (VMD) [38]; this technique subdivides the signal into *N* intrinsic mode functions, each having a frequency spectrum centered around a central frequency. The number of intrinsic mode functions was set to 3, with the high- and mid-central frequencies (f1 and f2, respectively) assumed to be potential descriptors of wobbling or involuntary arm swing artifacts and the low-central frequency (f3) associated with the jump itself [29].

### 2.4. Model Creation and Evaluation

After data cleaning, 286 out of 342 jumps were available for the definition of the final dataset. The discarded jumps were affected by one of these two issues: lack of synchronization between signals coming from gyroscope and accelerometer or abnormal drift amplitude in the acceleration signal, eventually occurring in a single testing session with many participants due to SP overuse.

The final dataset of 286 jumps was made available, each leading to a record including the abovementioned 61 features computed from a_V_ and a_AP_, as well as the SLJ length, l_meter_, taken from the meter tape and considered as the dependent variable. Once data were arranged for all the jumps, the dataset was separated into two subsets: 80% of the jumps (229 examples) was used as training set, and the remaining 20% (57 examples) was used as test set. This separation was entrusted to a randomization algorithm. Before training, z-score was used to normalize each feature of the training set [39]. The test set was normalized with normalizing factors taken from the training set. Lasso regularization was used on the training set to perform a feature reduction in order to avoid possible multicollinearity among features [40], choosing α = 0.1 to set the regularization strength. The features that were excluded by such a shrinkage were not used to develop the ML models.

The following regression models were trained using the MATLAB Regression Learner app (MATLAB and Statistics and Machine Learning Toolbox™ R2022a, The MathWorks, Inc., Natick, MA, USA): linear regression (LR) and stepwise regression (SR); optimized support vector machines (SVMs); optimized ensemble; optimized gaussian process regression (GPR) models; optimized neural networks (NNs). The optimized models were obtained using Bayesian optimization criterion [41] limiting the number of iterations to 30. The models were trained using a 10-fold cross validation procedure to stress model generalizability. For each trained model, the root-mean-square error (RMSE), mean squared error (MSE), mean absolute error (MAE), and R^2^ were computed for both training and test sets using the Regression Learner MATLAB app. The list of optimized hyperparameters using the Bayesian optimization method is reported in Table 2 for each selected architecture.

After training, the best model for each architecture (Table 3) was selected based on the minimum RMSE and used in the successive test phase.

The best model across all architectures was identified and used on the training set to analyze how much each input variable influences the estimate and enriches model interpretability. To this aim, permutation feature importance (PFI) analysis [42,43,44] was performed on the training set. PFI is an iterative process based on the analysis of the model error, evaluated as mean squared error (MSE), in output from the model when one of the input variables is randomly permuted and the others are maintained as they are. This analysis is performed for each input variable and provides an index of their importance, computed as the ratio between the MSE obtained by the permutation of the i-th input variable (MSE_i_) and the MSE of the model without any permuted variables (MSE_0_). The higher the ratio, the higher the contribution of the i-th variable to the estimate, and vice versa.

### 2.5. Statistical Analysis

The best model for each architecture was analyzed on the test data using Bland and Altman plots [45]. The upper limit (UL) and lower limit (LL) were calculated, respectively, as follows: UL = BIAS + 1.96 × SD; LL = BIAS − 1.96 × SD (BIAS = test value—model predicted value; SD = standard deviation of the previous differences). Moreover, confidence intervals (CI) at 95% of BIAS, UL, and LL were calculated as the following [46]: t-value, number of samples in test set (n), and standard error for the BIAS (SE_BIAS_) used of CI calculations and were reported in Table 4. Confidence intervals, as well as the regression line of the averages vs. differences, characterized by the coefficient and intercept value and the associated $R_{AB}^{2}$, were reported in Bland and Altman plots. The Kendall’s τ coefficient [47] was calculated to verify the presence of data heteroscedasticity.

Moreover, the models’ performances were evaluated using three metrics applied on the test set: (i) accuracy, obtained as the RMSE between the reference value and the estimated one; (ii) precision, obtained as the standard deviation of the distance between the reference and estimated values; (iii) bias, obtained as the mean distance between the reference values and the estimated ones.

## 3. Results

The 286 jumps had a l_meter_ of 1.83 ± 0.30 m, ranging from 1.12 m to 2.60 m.

After Lasso regularization, 11 out of 61 features were used to train the ML models. The best models obtained after Bayesian optimization for each architecture along with their optimized hyperparameters are reported in Table 3.

**Figure 3:**
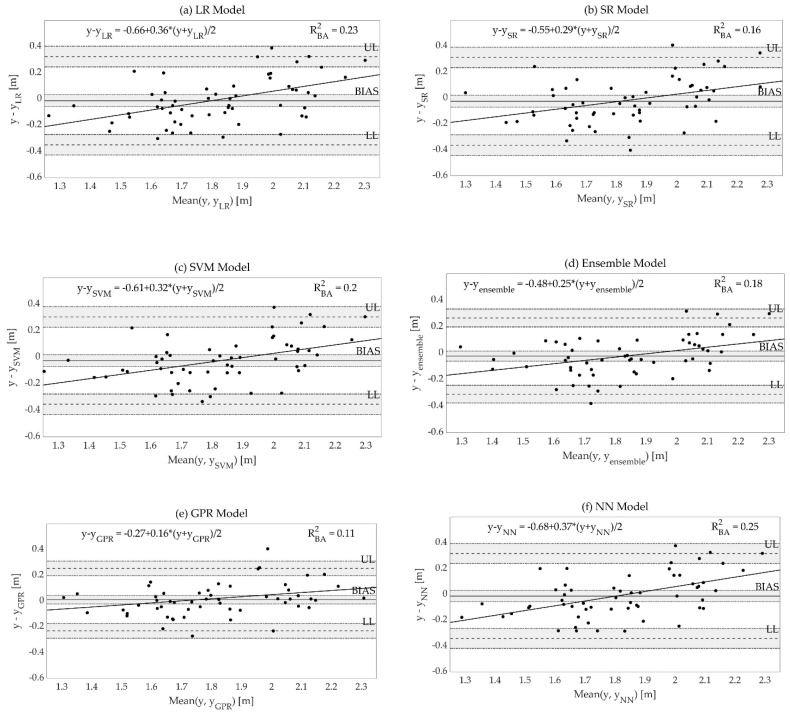
Bland–Altman plots of the test set for: (**a**) linear regression (LR) model; (**b**) stepwise regression (SR) model; (**c**) SVM model; (**d**) ensemble model; (**e**) GPR model; (**f**) neural network (NN) model. For each model, the mean (BIAS), upper limit (UL), and lower limit (LL) of the difference are reported. Average and difference are computed in meters using the test set, where y = reference output of test data, l_meter_, and y_i_ = estimated output of the i-th trained model. In grey, confidence intervals are reported for BIAS, UL, and LL. The regression line is reported in black; the regression equation and the associated R_BA_^2^ are reported on the top of the plots.

The Bland and Altman plots relative to the models listed in Table 3 are reported in Figure 3.

The metrics of the best models together with the values of bias, UL, and LL of the Bland and Altman plots are reported in Table 4.

**Figure 4:**
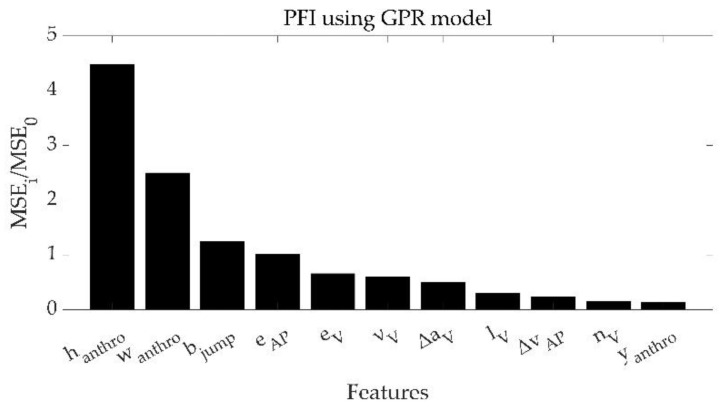
Results of PFI analysis performed on the variables used to train the best model (GPR).

Among the optimized models, the GPR model presented the best performances, with the highest R^2^ (0.81) as well as the best values for all the analyzed performance metrics. The model was homoscedastic (τ = 0.06). Referring to this model, in Figure 4, the list of key features is reported and sorted based on their importance as assessed by the PFI score.

## 4. Discussion

In this study, the SLJ distance was estimated investigating the use of low-cost IMUs, as available in current smartphones, in combination with different machine learning architectures. The protocol proposed in this work is designed for an ecological setting and an unsupervised user administration: by extracting features only in the preparation phase and holding the smartphone with the hand at the hip level, robust estimates of the SLJ distance are allowed.

The GPR model proved to be the most capable at describing the jumped distance variation in training (R^2^ = 0.88) and test set (R^2^ = 0.81), while presenting the best accuracy (12 cm, 6.6% of the jumped length) among all the trained architectures. This choice also grants better precision and bias (12 and 1 cm, respectively), as well as estimation errors independent from the magnitude of the jumped distance, as detailed in Table 4 and observed in the relevant Bland–Altman plot. Ninety-six percent of the tested jumps had an absolute error below 15%, spanning from a minimum error (underestimation) of −18.5% to a maximum error (overestimation) of 17.0%. Given the error obtained, we could speculate that it is least problematic when performing an analysis where a high variability is expected such as in children’s motor development; however, it remains within the subjectivity of the tester whether such error is considered acceptable or not in other applicative contexts.

These results constitute an improvement with respect to the only available model for SLJ distance estimate [2], which presented an RMSE of 15.4 cm. This improvement can be attributed to the use of features related to the biomechanics of the jump which allows estimating the SLJ distance differently for each jump of the same person. Conversely, the model by Akay and co-authors is not able to capture intra-individual variations of the SLJ length since it only includes anthropometric variables and the sport branch as input variables. Moreover, the population sampled in [2] was younger (9 to 13 years old), limiting the length of the analyzed jump and the generalizability of the model to jumpers of similar age.

Lasso regularization was used as a tool for removing multicollinear, redundant features. Overall, 11 out of 61 features were selected for model training: 3 of them are related to the participants anthropometric characteristics, while the remaining ones come from both the AP and V components. This suggests that both directions contribute to the overall performance of the test.

For the proposed model and following the PFI analysis, the features that mostly affect the results if permutated (MSE ratio > 1, Figure 4) are the height and the body mass of the participant (h_anthro_ and w_anthro_, respectively), the distance jumped estimated under the ballistic motion assumption (b_jump_), and the maximum acceleration in the antero-posterior direction (e_AP_). While on the one hand, the role of the anthropometric features in the estimate of the SLJ length confirms Akay’s results [2], on the other hand, the presence of b_jump_ confirms the important contribution to the estimate given by the ballistic motion hypothesis [48]. Noteworthily, b_jump_ cannot be considered alone for the SLJ length estimate (thus using only IMUs information without the use of ML approaches), as it leads to a MAE on the test set of about 0.57 m, underperforming the current results using the GPR model (0.09 m as in Table 3). Finally, the presence of e_AP_ as a predictive feature confirms that the power production in the anterior–posterior direction is crucial in the SLJ distance, consistently with the use of this measure as a test to assess power [7,10,11,49].

The results presented here should be evaluated within the following limitations: (i) the reported quality can be expected only when the proposed model is applied to jumps within the same range of those measured (1.12–2.60 m); (ii) the model applies to jumps performed with the hands at hip level, therefore limiting the analysis to the role of the lower limbs alone, and thus neglecting the theoretical positive contribution given by the upper limbs. Moreover, we anecdotally experienced that the prolonged use of the smartphone in the same session caused the random loss of signal. This problem may depend on several SP factors whose investigation is outside the scope of this research, but are worth keeping in mind for in-the-field use of this research.

Finally, regarding the strong point of this approach, we can highlight the following important aspects: (i) while the proposed method only provides the SLJ distance, the adoption of IMUs gives access to biomechanical features, thus offering the opportunity to analyse jumping technique of the athlete; (ii) the proposed integrated approach (IMU data + machine learning) outperforms SLJ length estimates obtained using only IMUs; (iii) the proposed method is objective and ecologic as it could be fully applied in the sport field using only SPs in stand-alone modality, i.e., without the need to involve external testers, thus enabling self-monitoring applications.

In the future, such insights could be derived through a dedicated app, allowing easy and widespread access to this information. Of particular interest would be predicting peak and average power as attempted for CMJ [30,50,51] and SLJ [11]. Specifically, this latter study used both SLJ distance and anthropometric variables as predictors of peak and average power. In this perspective, a future step could be to improve the models proposed by Mann [11] with the integration of biomechanical features such as those included in the current study. The exploratory work by Harry, evaluating the potential contribution of variables derived from force plates to SLJ power production, could also set the stage for expanding the current work.

## 5. Conclusions

This study proved the feasibility of using low-cost smartphone sensors to provide an automatic and objective estimate of SLJ performance in ecological settings. This result was made possible by complementing smartphone-based measurement with state-of-the-art machine learning methods. Based on the massive use of jumping testing in team sports and the wide availability of smartphones, it is believed that such a democratic approach could represent an added value in players’ assessment, especially when working at the amateur level.

## Figures and Tables

**Table 1:** List of the selected features reported with their acronym (ID), measurement unit and brief description. V and AP subscripts are relative to vertical and anteroposterior components of velocity/acceleration. The superscript * is used when the features are extracted from both AP and V components. Anthropometric features are reported with (·)_anthro_ subscript. Capital letters are for time intervals, small letters for the other features. Features are grouped by type and ordered following the alphabet. Legend: a.u. = arbitrary units; instants of: t_0_ = jump onset; t_a_min_ = minimum acceleration; t_a_max_ = maximum acceleration; t_v_min_ = minimum velocity; t_P_min_ = minimum power; t_P_max_ = maximum power; t_TO_ = jump take off, t_UL_ = jump unloading, t_BP_ = jump braking.

	ID	Feature	Measurement Unit	Description
**Anthro**	h_anthro_	Stature of the participant	m	-
	w_anthro_	Body mass of the participant	kg	-
	y_anthro_	Age of the participant	y	-
**Ballistic**	α	Velocity angle at take off	deg	$\alpha =\mathrm{arctg}\left(\frac{v_{V}\left(t_{\mathrm{TO}}\right)}{v_{\mathrm{AP}}\left(t_{\mathrm{TO}}\right)}\right)$
	b_jump_	Ballistic SLJ length	m	$b_{\mathrm{jump}}=\left(\frac{2{\;v}_{V}\left(t_{\mathrm{TO}}\right){\;*\;v}_{\mathrm{AP}}\left(t_{\mathrm{TO}}\right)}{g}\right)$
	h_jump_	Ballistic SLJ height	m	$h_{\mathrm{jump}}=\frac{{\left(v_{V}\left(t_{\mathrm{TO}}\right)\right)}^{2}}{2\;*\;g}$
	t_flight_	Ballistic time of flight	s	$t_{\mathrm{flight}}=\frac{2{\;*\;v}_{v}\left(t_{\mathrm{TO}}\right)}{g}$
**Biomechanical**	A _V_	Unweighting phase duration	s	[t_0_, t_UB_]
	b *	Minimum acceleration	m/s^2^	a_V_(t_a_^V^__min_)
	C *	Time from minimum to maximum acceleration	s	[t_a*_min_, t_a*_max_]
	Δa *	Range between min-to-max acceleration in the time between t_0_ and t_TO_	m/s^2^	${\Delta a}^{*}=\max\left(a^{*}\left(t_{0}÷t_{\mathrm{TO}}\right)\right)\;-\min\left(a^{*}\left(t_{0}÷t_{\mathrm{TO}}\right)\right)$
	Δv *	Range between min-to-max acceleration in the time between t_0_ and t_TO_	m/s	${\Delta v}^{*}=\max(v^{*}\left(\left(t_{0}÷t_{\mathrm{TO}}\right)\right)\;-\min\left(v^{*}\left(t_{0}÷t_{\mathrm{TO}}\right)\right)$
	D *	Main positive impulse time	s	Time duration of positive acceleration in a* signal in the time interval [t_0_, t_TO_]
	e *	Maximum acceleration	m/s^2^	a_V_(t_a_^V^__max_)
	F *	Time from acceleration positive peak to the take off	s	[t_a*_min_, t_TO_]
	G_V_	Ground contact duration	s	[t_0_, t_TO_]
	H *	Time from minimum acceleration to the end of the eccentric braking phase	s	[t_UL_, t_BP_]
	$i_{V}$	Maximum positive slope of a_V_	m/s^2^	$i_{V}=\max\left(\frac{d\left(a_{V}\left(t\right)\right)}{\mathrm{dt}}\right)\;t\in \;$[t_0_, t_BP_]
	J *	Time from the negative peak velocity to the end of the eccentric braking phase	s	[t_v*_min_, t_BP_]
	k *	Acceleration at the end of the eccentric breaking phase	m/s^2^	a*(t_BP_)
	l *	Negative peak power	W/kg	P(t_P*_max_)
	L_AP_	Power peaks delta time found in the range [t_0_ ÷ t_TO_]	s	[t_P_^AP^__min_, t_P_^AP^__max_]
	M *	Positive power duration in the V component	s	-
	n *	Positive peak power	W/kg	P(t_P*_min_)
**Biomechanical**	O *	Time distance between positive peak power and take-off	s	[t_P*_max_, t_TO_]
	p *	Mean slope between acceleration peaks	a.u.	$p^{*}=\frac{e^{*}-b^{*}}{C^{*}}$
	q *	Shape factor	a.u.	Ratio between the area under the curve from t_UB_ to the last positive sample prior t_TO_ (lasting D*) and the one of a rectangle of sides D* and e*
	Q_V_	Time duration between the eccentric braking phase and the take off	s	[t_BP_, t_TO_]
	r *	Impulse ratio	a.u.	$r^{*}=\frac{b^{*}}{e^{*}}$
	R_AP_	Entire positive power duration in the AP component	s	-
	u *	Mean concentric power	W/kg	Average value of P*(t), t ∈ [t_BP_, t_TO_]
	ν *	Minimum negative velocity	m/s	v*(t_v*_min_)
	W *	Power peaks delta time	s	[t_P*_min_, t_P_max_]
	z *	Mean eccentric power	W/kg	Average value of P*(t), t ∈ [t_0_, t_BP_]
**Time-frequency**	f1 *	High central frequency	Hz	Highest VMD central frequency, associated with wobbling and noise
	f2 *	Middle central frequency	Hz	Middle VMD central frequency, associated with wobbling tissues
	f3 *	Low central frequency	Hz	Lower VMD central frequency, associated with the jump proper

**Table 2:** Type of selected models used for regression. The first column reports the architecture; the second reports the hyperparameter options for each architecture that can be personalized or optimized in the Regression Learner app; the third reports the ranges of hyperparameters optimization (using the default values in MATLAB).

Model	Hyperparameter	Hyperparameter Options/Ranges
Linear regression (LR)	-	-
Stepwise regression (SR)	-	-
SVMs	Function	Gaussian, Quadratic, Cubic, Linear
	Epsilon	[3.15 × 10^−4^, 31.50]
	Box Constraint	[10^−3^, 103]
	Kernel Scale	[10^−3^, 103]
Ensemble	Function	Bag, LSBoost
	Minimum leaf size	[1, 114]
	Number of learners	[10, 500]
	Number of predictors to sample	[1, 11]
GPR	Function	Rational Quadratic, Exponential, Matern 5/2, Matern 3/2, Squared Exponential
	Sigma	[10^−4^, 3.05]
	Basis Function	Constant, Zero, Linear
NNs	Function	Sigmoid, Tanh, ReLu, None
	Number of connected layers	[1, 3]
	Layer size	[1, 300]
	Lambda	[4.36 × 10^−8^, 4.36 × 10^2^]

**Table 3:** List of the best models for each architecture selected considering the model with the lowest RMSE value (train test). Models are reported along with their functions and optimized hyperparameters. Legend: * refers to a kernel function; ^§^ refers to an activation function.

Model	Function	Optimized Hyperparameters	RMSE [m]	MSE [m^2^]	MAE [m]	R^2^
LR		-	0.18–0.17	0.03–0.03	0.14–0.14	0.67–0.63
SR		-	0.19–0.17	0.04–0.03	0.15–0.14	0.60–0.62
SVMs	Gaussian *	Box Constraint: 0.7205 Epsilon: 0.07	0.18–0.18	0.03–0.03	0.14–0.14	0.66–0.59
Ensemble	-	Learners: 70 Minimum leaf size: 1 Predictors to sample: 11 Method: Bag	0.16–0.15	0.03–0.02	0.13–0.12	0.73–0.72
GPR	Rational Quadratic *	Sigma: 1.949 × 10^−4^ Basis Function: Linear	0.11–0.12	0.01–0.02	0.08–0.09	0.88–0.81
NNs	Sigmoid ^§^	Fully connected layers: 1 Lambda: 0.0116 Layer size: 1	0.17–0.17	0.03–0.03	0.14–0.14	0.68–0.64

**Table 4:** Metrics for each model: accuracy, precision, bias, UL, and LL of the difference are expressed in meters. Kendall’s tau coefficient (τ) is used to infer about data homoscedasticity (τ < 0.1). Samples (n); CI = confidence interval; t-value; SE_BIAS_ = standardized error of the estimates are also presented.

Parameter	LR	SR	SVMs	Ensemble	GPR	NNs
Accuracy [m]	0.17	0.17	0.18	0.15	0.12	0.17
Precision [m]	0.17	0.17	0.18	0.15	0.12	0.17
Bias [m]	−0.01	−0.03	−0.04	−0.02	0.01	−0.01
CI_BIAS_ (95%) [m]	[−0.06, 0.03]	[−0.07, 0.02]	[−0.07, 0.02]	[−0.06, 0.02]	[−0.02, 0.04]	[−0.06, 0.03]
UL [m]	0.32	0.31	0.31	0.027	0.25	0.32
CI_UL_ (95%) [m]	[0.25, 0.40]	[0.23, 0.39]	[0.23, 0.38]	[0.19, 0.33]	[0.19, 0.31]	[0.24, 0.39]
LL [m]	−0.35	−0.36	−0.36	−0.31	−0.23	−0.34
CI_LL_ (95%) [m]	[−0.43, −0.27]	[−0.44, −0.28]	[−0.43, −0.23]	[−0.38, −0.24]	[−0.29, −0.17]	[−0.42, −0.26]
Kendall’s τ	0.06	0.021	0.02	0.08	0.06	0.06
Samples (n)	57	57	57	57	57	57
t-value	2.00	2.00	2.00	2.00	2.00	2.00
SE_BIAS_ (s/√n)	0.02	0.02	0.02	0.01	0.01	0.02

## Data Availability

The raw data supporting the conclusions of this article will be made available by the authors, without undue reservation (https://github.com/BeatriceDL?tab=repositories, accessed on 26 April 2023).
